# Dimensional association between ADHD traits and gray matter volume in young adults: A voxel‐based morphometry study

**DOI:** 10.1002/pcn5.70325

**Published:** 2026-03-31

**Authors:** Haruka Asaoka, Michio Takahashi, Kentaro Oba, Hikaru Takeuchi, Yasuyuki Taki

**Affiliations:** ^1^ Department of Aging Research and Geriatric Medicine, Institute of Development, Aging and Cancer Tohoku University Sendai Japan; ^2^ Smart Aging Research Center Tohoku University Sendai Japan; ^3^ Division of Developmental Cognitive Neuroscience, Institute of Development, Aging and Cancer Tohoku University Sendai Japan

**Keywords:** ADHD traits, attention‐deficit/hyperactivity disorder (ADHD), dimensional approach, gray matter volume, voxel‐based morphometry (VBM)

## Abstract

**Aim:**

Prior structural magnetic resonance imaging (MRI) studies of attention‐deficit/hyperactivity disorder (ADHD) have primarily focused on clinically diagnosed samples, whereas the neuroanatomical correlates of dimensional ADHD traits in healthy young adults (aged 18–27 years) remain insufficiently characterized, and it is unclear whether neuroanatomical patterns reported in clinically diagnosed samples generalize to subclinical trait variability in young adulthood. We therefore examined whole‐brain voxelwise associations between gray matter volume (GMV) and the Conners' Adult ADHD Rating Scales (CAARS) Hyperactivity/Restlessness subscale as the primary analysis, and evaluated associations with other CAARS scales on an exploratory basis, using voxel‐based morphometry (VBM).

**Methods:**

Participants were 534 healthy young adults (304 males, 230 females; aged 18–27 years) without an ADHD diagnosis. ADHD traits were assessed using CAARS. High‐resolution T1‐weighted structural MRI data were acquired on a 3‐Tesla scanner. GMV was analyzed using VBM implemented in SPM12. Voxelwise multiple‐regression analyses were conducted, with Hyperactivity/Restlessness as the primary scale of interest and other CAARS scales examined in separate exploratory models. Statistical significance was set at *p* < 0.05 (cluster‐level family‐wise error [FWE]‐corrected), using a voxelwise cluster‐forming threshold of *p* < 0.001 (uncorrected).

**Results:**

GMV in the right dorsolateral prefrontal cortex (DLPFC) showed a small but statistically significant positive association with Hyperactivity/Restlessness scores (cluster‐level FWE‐corrected *p* = 0.017). No significant associations were observed for other CAARS subscales, and no significant sex‐stratified effects were detected.

**Conclusion:**

These findings are consistent with a dimensional perspective on ADHD‐related traits and suggest that interindividual differences in Hyperactivity/Restlessness may be reflected in subtle GMV variation in the right DLPFC among healthy young adults. Further multimodal and longitudinal studies are warranted to clarify developmental and mechanistic interpretations.

## Graphical abstract

This study examined the association between attention‐deficit/hyperactivity disorder (ADHD) traits and gray matter volume (GMV) in 534 healthy young adults. Higher Hyperactivity/Restlessness scores were associated with greater GMV in the right dorsolateral prefrontal cortex (DLPFC), a region implicated in executive control and behavioral regulation. No significant associations were found for other Conners' Adult ADHD Rating Scales (CAARS) subscales or in sex‐stratified analyses. These findings support a dimensional view of ADHD‐related traits.

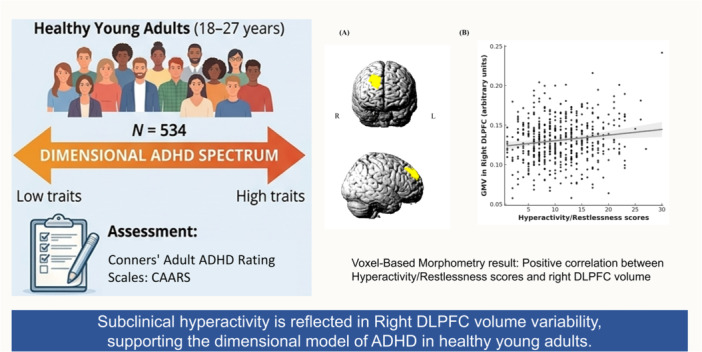

## INTRODUCTION

The diagnostic framework of attention‐deficit/hyperactivity disorder (ADHD) has long been debated, particularly regarding the validity of categorical versus dimensional approaches.^1^, ^2^, ^3^, ^4^ Whereas the Diagnostic and Statistical Manual of Mental Disorders, Fifth Edition, Text Revision (DSM‐5‐TR) defines ADHD categorically based on symptom thresholds, dimensional models conceptualize ADHD‐related traits as continuously distributed in the general population.^5^ Consistent with this dimensional perspective, ADHD‐related traits vary widely even among individuals without a clinical diagnosis, and such subclinical variability may be associated with functional difficulties.^6^, ^7^, ^8^, ^9^, ^10^

Neuroimaging studies have consistently shown that ADHD is associated with structural alterations in gray matter volume (GMV), particularly in regions such as the prefrontal cortex, basal ganglia, and cerebellum—components of the fronto‐striatal‐cerebellar network.^11^, ^12^, ^13^ Across studies, reported GMV differences have varied by brain region and developmental stage.

However, most evidence has come from case–control studies in clinically diagnosed children and adolescents, and voxelwise evidence on the structural correlates of dimensional ADHD traits in young adulthood remains limited.^14^, ^15^ Young adulthood (aged 18–27 years) is characterized by increasing academic and social demands, making it a relevant developmental stage for examining how individual differences in attention and behavioral regulation relate to functional outcomes.^16^, ^17^ Therefore, we examined whole‐brain voxelwise associations between GMV and ADHD‐related traits assessed using the Conners' Adult ADHD Rating Scales (CAARS) in a nonclinical sample of healthy young adults using voxel‐based morphometry (VBM), with particular interest in Hyperactivity/Restlessness. Associations with other CAARS scales and sex‐stratified analyses were conducted on an exploratory basis.

## METHODS

### Participants

Structural magnetic resonance imaging (MRI) and questionnaire data were drawn from a large‐scale neuroimaging project conducted at the Institute of Development, Aging and Cancer, Tohoku University.^18^ Participants were recruited via university bulletin boards, email announcements, and local media advertisements. Exclusion criteria included metal implants, pregnancy, claustrophobia, regular use of prescription medication, a history of head trauma, and current or past neurological or psychiatric disorders (e.g., depression, anxiety disorders, autism spectrum disorder, learning disabilities, and epilepsy). Handedness was assessed using the Edinburgh Handedness Inventory,^19^ and only right‐handed participants were included. The initial dataset comprised 1770 healthy young adults (Japanese university students) aged 18–27 years. Of these, 600 participants completed the CAARS; those without CAARS data (*n* = 1170) were excluded. Two participants were excluded based on MRI quality control (e.g., gross artefacts and/or segmentation failure on visual inspection). To ensure response validity, we excluded individuals with elevated CAARS validity indices (Inconsistency Index ≥ 8, *n* = 55; Infrequency Index ≥ 21, *n* = 9), yielding a final analytic sample of 534 participants (Table 1). Because CAARS was administered to only a subset of the original cohort, the analytic sample may not be fully representative of the initial recruitment pool. Written informed consent was obtained from all participants. For participants younger than 20 years, written consent was also obtained from legal guardians.

**Table 1:** Participant demographics and Conners' Adult ADHD Rating Scales (CAARS) scores.

Measure	Mean ± SD	Min–Max
Age	20.9 ± 1.6	18–27
Sex		–
Male	304	
Female	230	
CAARS subscales		
Inattention/Memory Problems	13.8 ± 6.1	0–31
Hyperactivity/Restlessness	10.8 ± 5.4	1–30
Impulsivity/Emotional Lability	10.4 ± 5.8	0–34
Problems with Self‐Concept	8.8 ± 4.2	0–18
ADHD Index	7.7 ± 4.5	0–24
DSM‐IV Inattentive Symptoms	5.8 ± 4.0	0–19
DSM‐IV Hyperactive‐Impulsive Symptoms	13.6 ± 7.7	0–36
DSM‐IV Total ADHD Symptoms	11.5 ± 5.3	0–28

*Note*: The demographic data, along with the mean scores and standard deviations for the CAARS factor‐derived subscales, ADHD Index, and DSM‐IV‐based ADHD symptom scales, are summarized for the full sample (*N* = 534).

Abbreviation: ADHD, attention‐deficit/hyperactivity disorder.

### Assessment of ADHD traits

ADHD traits were assessed using the Japanese version of the CAARS, which has been standardized and validated for the Japanese population.^20^ The 66‐item self‐reported form was administered. This scale includes four empirically derived factor subscales—Inattention/Memory Problems (12 items), Hyperactivity/Restlessness (12 items), Impulsivity/Emotional Lability (12 items), and Problems with Self‐Concept (6 items)—as well as three DSM‐IV‐based subscales (DSM‐Inattentive Symptoms, DSM‐Hyperactive‐Impulsive Symptoms, and DSM‐Total ADHD Symptoms). Additionally, the ADHD Index (12 items) and the Inconsistency Index (16 items) were used to assess ADHD‐related behaviors and response consistency, respectively. Items were rated on a 4‐point Likert scale (0 = not at all true, 3 = very much true). Voxelwise analyses were conducted using raw sum scores for each CAARS subscale. For each subscale, raw sum scores were calculated. Individuals with an Inconsistency Index score ≥ 8 were excluded due to concerns regarding response reliability.

### MRI acquisition

Structural MRI data were acquired using a 3‐Tesla Philips Achieva Quasar Dual scanner (Philips Medical Systems, the Netherlands) equipped with an 8‐channel head coil. A magnetization‐prepared rapid gradient echo sequence optimized for structural imaging was used. The imaging parameters were as follows: matrix size = 240 × 240; repetition time = 6.5 ms; echo time = 3 ms; inversion time = 711 ms; field of view = 240 mm; number of slices = 162; slice thickness = 1.0 mm; and scan duration = 8 min and 3 s.

### Image preprocessing

Structural MRI data were analyzed using brain VBM implemented in SPM12 (Statistical Parametric Mapping, Wellcome Department of Imaging Neuroscience, London, UK) and MATLAB R2022b (MathWorks, Natick, MA, USA). Image preprocessing was conducted as follows. All structural images were manually reoriented to align with the anterior commissure–posterior commissure line. Tissue segmentation was performed to classify the voxels into six tissue classes: gray matter, white matter, cerebrospinal fluid, skull, extracranial soft tissue, and air. Voxels with a combined gray and white matter signal intensity of less than 0.25 were excluded to avoid misclassification of nonbrain tissues. For spatial normalization, Diffeomorphic Anatomical Registration Through Exponentiated Lie Algebra was applied using a template specific to East Asian populations, and images were transformed into Montreal Neurological Institute (MNI) space. All images were smoothed with an 8‐mm FWHM Gaussian kernel to improve sensitivity to spatially extended effects. We note that cluster‐level inference can be influenced by analytic choices such as the smoothing kernel and the cluster‐forming threshold.

### Statistical analysis

Statistical analyses were performed within a general linear model framework implemented in SPM12. Whole‐brain voxelwise multiple‐regression analyses were conducted with voxelwise GMV as the dependent variable.

The primary analysis tested the association between GMV and the CAARS Hyperactivity/Restlessness subscale. Secondary exploratory analyses examined associations with the remaining CAARS factor‐derived subscales, DSM‐IV‐based symptom scales, and the ADHD Index, using separate regression models for each scale. Given the number of behavioral scales examined, these secondary analyses were considered hypothesis‐generating, and no additional correction was applied across behavioral measures.

In each model, the CAARS scale of interest was entered as the predictor along with predefined covariates (age and total brain volume [TBV]). CAARS scales were not entered simultaneously in a single model because these measures are intercorrelated, and simultaneous entry could introduce multicollinearity and hinder interpretability. In our sample, CAARS subscales showed substantial intercorrelations, and when the eight CAARS scales were entered simultaneously, variance inflation factors indicated severe multicollinearity (e.g., variance inflaction factors for DSM‐IV Inattentive Symptoms, DSM‐IV Hyperactive‐Impulsive Symptoms, and DSM‐IV Total ADHD Symptoms were 983.77, 786.20, and 2886.95, respectively). This was expected because DSM‐IV Total ADHD Symptoms is a composite of the DSM‐IV Inattentive and Hyperactive‐Impulsive symptom counts. Therefore, each CAARS scale was evaluated in a separate regression model.

Voxelwise statistical maps were thresholded at *p* < 0.001 (uncorrected) to define clusters, and statistical significance was assessed at *p* < 0.05 using cluster‐level family‐wise error (FWE) correction.

## RESULTS

### VBM results

**Figure 1:**
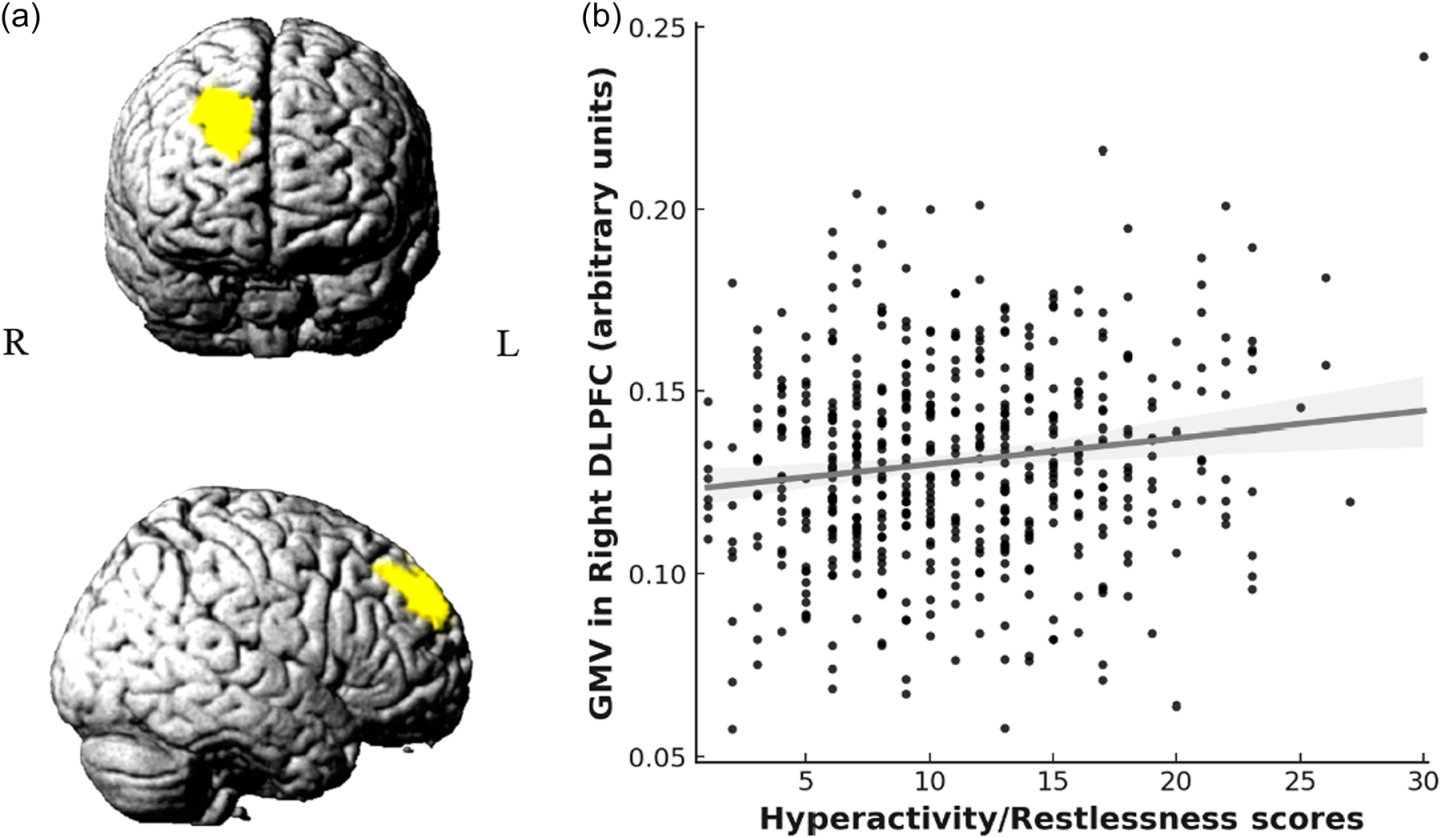
(A) Brain region showing a significant positive correlation between gray matter volume (GMV) and Hyperactivity/Restlessness subscale scores (highlighted in yellow), identified using whole‐brain voxel‐based morphometry (VBM) analysis (cluster‐level *p* < 0.05, family‐wise error [FWE]‐corrected). The brain image includes orientation labels (L = left, R = right). (B) Scatter plot illustrating the positive correlation between GMV values extracted from the peak voxel (Montreal Neurological Institute [MNI] coordinates: 18, 51, 29) of this cluster in the right dorsolateral prefrontal cortex (DLPFC) and Hyperactivity/Restlessness scores. Each dot represents one participant.

The primary whole‐brain VBM analysis revealed a statistically significant positive association between CAARS Hyperactivity/Restlessness scores and GMV in the right dorsolateral prefrontal cortex (DLPFC), surviving cluster‐level FWE correction (voxelwise *p* < 0.001, cluster‐level FWE‐corrected *p* < 0.05; *p* = 0.017; Table 2). As shown in Figure 1, higher Hyperactivity/Restlessness scores were associated with greater GMV in the right DLPFC, with a modest positive correlation in the extracted cluster GMV (*r* = 0.143, 95% CI [0.059, 0.225], *p* = 0.001). In secondary exploratory analyses, no significant whole‐brain associations were observed for the other CAARS subscales, including Inattention/Memory Problems and Impulsivity/Emotional Lability. Exploratory sex‐stratified analyses likewise did not reveal any significant associations in either males or females at the whole‐brain level (voxelwise *p* < 0.001, uncorrected; cluster‐level FWE‐corrected *p* < 0.05).

**Table 2:** Significant clusters showing associations between gray matter volume (GMV) and Hyperactivity/Restlessness scores (whole‐brain voxel‐based morphometry [VBM] analysis).

CAARS subscale	Brain region	MNI (*x*, *y*, *z*)	Peak T		Cluster extent (voxels)	*p* (FWE‐corr)
Hyperactivity/Restlessness	Dorsolateral prefrontal cortex (DLPFC)	18, 51, 29	4.33		1643	0.017

*Note*: Only clusters surviving whole‐brain correction for multiple comparisons (voxelwise *p* < 0.001, uncorrected; cluster‐level FWE‐corrected *p* < 0.05) are reported. Peak T denotes the maximum *t* statistic within each cluster at the reported MNI coordinates. No significant associations were found for other CAARS subscales (Inattention/Memory Problems, Impulsivity/Emotional Lability) or in sex‐stratified analyses.

Abbreviations: CAARS, Conners' Adult ADHD Rating Scales; FWE, family‐wise error; MNI, Montreal Neurological Institute.

## DISCUSSION

Accumulating evidence supports a dimensional approach for understanding ADHD, in which trait‐level symptoms are conceptualized as continuously distributed across individuals. The present study examined voxelwise associations between ADHD‐related traits and GMV in healthy young adults. The primary finding was a statistically significant positive association between CAARS Hyperactivity/Restlessness scores and GMV in the right DLPFC, specifically within the superior frontal gyrus. In secondary exploratory analyses, no significant whole‐brain associations were observed for the other CAARS subscales, and exploratory sex‐stratified analyses likewise did not yield significant effects.

Although the present findings demonstrated a positive association between GMV in the right DLPFC and Hyperactivity/Restlessness traits, previous neuroimaging studies have predominantly reported GMV reductions in children and adolescents with ADHD, particularly within the prefrontal cortex, cerebellum, hippocampus, and amygdala.^21^, ^22^, ^23^ Nevertheless, increased GMV has also been observed in the primary motor cortex, supplementary motor area, DLPFC, inferior parietal lobule, and orbitofrontal cortex,^24^, ^25^, ^26^, ^27^, ^28^ with the current results aligning with this latter body of evidence. Extending these categorical clinical findings to a nonclinical cohort of healthy young adults, the present dimensional analysis indicates that interindividual variability in ADHD‐related traits covaries with right DLPFC GMV, with the association most evident for Hyperactivity/Restlessness.^29^, ^30^ These results suggest that DLPFC enlargement is not restricted to case‐defined ADHD samples and may also be detectable as subtle trait‐related neuroanatomical variability within the general population.

In addition, most prior neuroimaging studies have focused on clinically diagnosed ADHD populations characterized by greater symptom severity and frequent pharmacological treatment.^12^, ^31^ In contrast, the present study investigated a nonclinical cohort of unmedicated healthy young adults (aged 18–27 years) recruited from a university setting. While previous studies have reported increased GMV in the prefrontal cortex and basal ganglia following stimulant medication,^12^, ^31^ the unmedicated status of the present sample suggests that the observed DLPFC enlargement is unlikely to be attributable to pharmacological neuroplasticity. In this nonclinical cohort of healthy young adults, the observed association was specific to Hyperactivity/Restlessness and should be interpreted as reflecting subclinical trait variability rather than subthreshold ADHD in a broad clinical sense. … Although participants were unmedicated, these data do not provide direct evidence for neurodevelopmental mechanisms. However, this cross‐sectional study cannot distinguish among alternative non‐pharmacological explanations, and unmeasured factors (e.g., sleep, physical activity, stress, cognitive ability, and socioeconomic variables) may also contribute to GMV variation.

With respect to interpretation, because this study is cross‐sectional, it cannot address developmental trajectories or causal relationships. Therefore, this association in healthy young adults should not be taken as evidence for delayed cortical maturation, which is primarily supported by longitudinal developmental trajectories in childhood and adolescence.^29^, ^32^ Instead, the observed GMV variation in the DLPFC may reflect multiple processes that extend into young adulthood, including individual differences in cortical remodeling and experience‐dependent factors. Longitudinal and multimodal studies are needed to evaluate these developmental accounts. The DLPFC is a core region for executive control and behavioral inhibition, and functional MRI studies have consistently reported altered or trait‐related DLPFC activation during inhibitory control paradigms in relation to Hyperactivity/Restlessness.^33^ Although structural and functional findings are not directly interchangeable, our result is broadly compatible with this functional literature implicating the DLPFC in hyperactivity‐related phenotypes in the general population.^33^

We found no significant associations between inattention traits and GMV. This finding is consistent with previous reports that also failed to identify significant associations between inattention and regional GMV in adults with ADHD.^34^ However, because the present study used structural MRI alone, mechanistic interpretations (e.g., delayed maturation or inhibitory‐control dysfunction) remain speculative and require confirmation using longitudinal and multimodal imaging (e.g., fMRI and diffusion MRI). Similar to these studies, the present sample of healthy young adults (aged 18–27 years) recruited from a university setting, in which inattention symptoms were relatively mild, may have reduced sensitivity to detect GMV correlates of inattention. Mechanistically, inattention may be more strongly associated with functional network‐level alterations—particularly within the default mode network (DMN) and fronto‐striatal circuitry—than with regional GMV differences detectable by VBM.^35^, ^36^, ^37^, ^38^ Aberrant DMN activity and impaired task‐related deactivation have been implicated in attentional lapses, a hallmark of inattention. Accordingly, static structural indices such as GMV may be insufficient to capture the neural underpinnings of inattention. Recent literature emphasizes that ADHD should be conceptualized not only as a disorder of localized abnormalities but also as one involving dysregulated connectivity across large‐scale brain networks.^37^, ^38^ From this standpoint, structural MRI alone may have limited sensitivity for detecting inattention‐related differences. Therefore, the null results should not be interpreted as evidence of a lack of a neurobiological basis but rather as indicative of mechanisms that may be primarily functional or network‐based. Future multimodal imaging studies are warranted to elucidate the neural substrates of inattention.

Contrary to our expectation, no significant associations were observed between sex and ADHD traits in predicting GMV. These findings align with previous evidence reporting limited sex differences in GMV among adults with ADHD, with effects largely confined to the caudate nucleus.^39^ Similarly, previous research reported GMV reductions in the basal ganglia in individuals with ADHD but did not provide a detailed analysis of sex differences.^12^

Several factors may account for the lack of sex‐specific effects. First, the current sample comprised healthy, unmedicated young adults exhibiting subclinical ADHD traits. The mild symptom levels and absence of pharmacological treatment may have contributed to minimal structural alterations, thus reducing the likelihood of detecting sex‐related effects. Second, sex differences in ADHD‐related brain morphology may be more pronounced earlier in development and may diminish or undergo reorganization in adulthood.^39^ Third, sex differences may not be reflected by gross volumetric measures such as GMV but may instead manifest in functional connectivity or microstructural properties.^38^, ^40^ Accordingly, the absence of significant sex differences in this study should not be construed as evidence for the absence of sex differences in the neurobiology of ADHD. Rather, it may reflect the complex interaction of symptom severity, developmental timing, and measurement sensitivity. Future research should employ multimodal imaging and longitudinal designs to clarify the contexts in which sex differences in ADHD emerge.

Several limitations of the present study should be acknowledged. First, the cross‐sectional design precludes causal inferences regarding the relationship between ADHD traits and brain structure. Longitudinal investigations are necessary to elucidate developmental trajectories in GMV associated with ADHD traits.

Second, ADHD traits were assessed using a self‐reported questionnaire, which may be subject to response biases, including underreporting or overreporting. Moreover, because ADHD traits were assessed only by self‐report (CAARS), the present study lacked informant ratings, clinical interviews, or objective behavioral measures, which may increase measurement bias and attenuate or distort brain–behavior associations. Future research should incorporate observer‐rated instruments or performance‐based behavioral assessments to enhance the reliability and objectivity of ADHD trait measurement.

Third, the sample consisted of healthy university students, potentially limiting the generalizability of the findings. The findings may not generalize to individuals with different educational backgrounds, cognitive characteristics, or socioeconomic contexts. Future studies should aim to include participants from a wider range of demographic and sociocultural backgrounds to improve external validity. In addition, the final analytic sample was derived from a larger cohort, and a substantial proportion of participants were excluded due to unavailable CAARS data and CAARS validity indices; therefore, selection bias cannot be ruled out. Moreover, the restriction to right‐handed Japanese university students may further limit generalizability.

Fourth, although key covariates such as age and TBV were statistically controlled, other relevant confounders—including intelligence quotient (IQ), academic achievement, sleep patterns, and socioeconomic indicators—were not measured. These unmeasured factors may be associated with both ADHD trait expression and GMV and therefore could partly account for the observed association, limiting the interpretability of the current results.

Finally, the study did not include retrospective or longitudinal data on childhood ADHD traits or neurodevelopmental history. Consequently, it was not possible to examine how the age of symptom onset or developmental course may be related to current brain structure. To clarify the long‐term neurodevelopmental implications of ADHD traits, future research should incorporate prospective longitudinal designs spanning from childhood to adulthood.

Given the number of behavioral measures examined, the possibility of inflated Type I error cannot be excluded, and the finding requires replication. Recent evidence suggests that brain–behavior associations can be unstable in moderate sample sizes, especially for small effect sizes, and that substantially larger samples may be needed to obtain reproducible estimates.^41^ Therefore, our modest association should be interpreted cautiously and requires replication in independent, larger cohorts.

## CONCLUSION

The present study investigated voxelwise associations between ADHD‐related traits and brain structure in healthy young adults using a dimensional neuroimaging approach. We identified a statistically significant, albeit modest, positive association between GMV in the right DLPFC and CAARS Hyperactivity/Restlessness scores.

These findings suggest that subclinical variability in Hyperactivity/Restlessness may be reflected in subtle GMV variation in the right DLPFC, providing preliminary support for a dimensional conceptualization of ADHD‐related traits in healthy young adults. Secondary analyses of other CAARS subscales, including DSM‐IV‐based subscales and the ADHD Index, were exploratory and did not yield significant whole‐brain associations. Future longitudinal and multimodal studies will be essential to clarify developmental and mechanistic interpretations. Such studies may also help identify potential moderators, including symptom severity, sex, and functional measures.

## AUTHOR CONTRIBUTIONS

**Haruka Asaoka**: Conceptualization; methodology; formal analysis; data curation; writing—original draft. **Michio Takahashi**: Investigation; methodology; writing—review and editing. **Kentaro Oba**: Validation; writing—review and editing. **Hikaru Takeuchi**: Investigation; funding acquisition. **Yasuyuki Taki**: Conceptualization; project administration; supervision.

## CONFLICT OF INTEREST STATEMENT

The authors declare no conflicts of interest.

## ETHICS APPROVAL STATEMENT

The study was approved by the Ethics Committee of the Graduate School of Medicine, Tohoku University (Approval Number: 2017‐87) and conducted in accordance with the Declaration of Helsinki.

## PATIENT CONSENT STATEMENT

Written informed consent was obtained from all participants.

## CLINICAL TRIAL REGISTRATION

N/A.

## Data Availability

The data that support the findings of this study are available on request from the corresponding author. The data are not publicly available due to privacy or ethical restrictions.
